# Evaluation of Full-Duplex SWIPT Cooperative NOMA-Based IoT Relay Networks over Nakagami-*m* Fading Channels

**DOI:** 10.3390/s22051974

**Published:** 2022-03-03

**Authors:** Tien-Tung Nguyen, Sang Quang Nguyen, Phu X. Nguyen, Yong-Hwa Kim

**Affiliations:** 1 Faculty of Electronics Technology, Industrial University of Ho Chi Minh City (IUH), Ho Chi Minh City 700000, Vietnam; nguyentientung@iuh.edu.vn; 2 Department of Science and International Cooperation, Ho Chi Minh City University of Transport, Ho Chi Minh City 700000, Vietnam; sang.nguyen@ut.edu.vn; 3 Department of Computing Fundamentals, FPT University, Ho Chi Minh City 700000, Vietnam; phunx4@fpt.edu.vn or; 4 Department of Data Science, Korea National University of Transportation (KNUT), Uiwang-si 16106, Gyeonggi-do, Korea

**Keywords:** NOMA, maximum ratio transmission, AF, outage probability, ergodic capacity

## Abstract

In this paper, we investigate the performance of non-orthogonal multiple access (NOMA)-based full-duplex Internet-of-Things (IoT) relay systems with simultaneous wireless information and power transfer (SWIPT) over Nakagami-*m* fading channels to improve the performance of a cell-edge user under perfect and imperfect successive interference cancellation (SIC). Two scenarios, i.e., direct and non-direct links, between the source node and cell-edge user are examined. The exact closed-form analytical and approximate expressions for the outage probability, system throughput, energy efficiency, and ergodic capacities are derived and validated via Monte Carlo simulations to characterize the proposed system performance. To further improve the system performance, we also provide a low-complexity algorithm to maximize the system throughput over-optimizing the time-switching factor. The results show that our proposed NOMA system can achieve superior performance compared to its orthogonal multiple access (OMA) counterpart under perfect SIC and with a low-to-medium signal-to-noise ratio under imperfect SIC, according to the level of residual self-interference and the quality of links.

## 1. Introduction

Recently, an exponential growth in the number of devices in the Internet of Things (IoT) network leads to massive connectivity and an increase in demand for spectrum usage [1,2,3]. Non-orthogonal multiple access (NOMA) has been recognized as a promising candidate for addressing the spectral efficiency (SE) problem, where NOMA is used to perform successive interference cancellation (SIC) and allow multiple users to have access to all resources in the power domain [4]. This is in contrast to most previous-generation technologies, which depend on the time, frequency, or code domain. On the other hand, radio frequency (RF) wireless energy harvesting (EH) has been considered as an effective solution to solve the problems of energy consumption [5,6,7]. Moreover, the technique was investigated in various systems such as multiple input multiple output (MIMO) network [8], secrecy network [9], unmanned aerial vehicle system [10,11], and Intelligent Reflecting Surface system [12].

Inspired by NOMA and RF-EH, the authors in [13] proposed a new kind of self-sustainable communication in B5G systems and IoT networks. The ergodic rate of a hybrid NOMA system with simultaneous wireless information and power transfer (SWIPT) has been investigated in [14]. By utilizing the majority of the received downlink power in the EH process, one can further improve the uplink rate of NOMA users. A hybrid user pairing scheme for improving spectral and energy efficiencies in multi-user multiple-input single-output SWIPT NOMA systems has been studied in [15]. The authors in [16] considered a bidirectional relaying SWIPT NOMA IoT relay system with perfect SIC (pSIC) and imperfect SIC (iSIC) to show that the proposed system attained throughput and capacity gains better than conventional bidirectional relaying multiple access schemes. SWIPT-enabled massive cellular NOMA IoT networks have been studied in [17], where the optimal solution for the spatial beam, transmit power, and power splitting coefficient were investigated to maximize the weighted sum rate and minimize the total power consumption under the impact of non-linear EH and imperfect SIC. The performance of hybrid EH in SWIPT NOMA for relaying networks was also investigated in [18], where the energy consumption with full channel state information at the transmitter (CSIT) and system outage probability (OP) with partial CSIT can be reduced significantly via the optimal time-switching coefficient.

Although NOMA has been considered a potential solution of SE, it is still subject to a serious issue wherein a user with strong channel conditions can intensify its capacity, while a user with weak channel conditions undergoes poor performance. With this in mind, a cooperative NOMA scheme has been proposed to reinforce the transmission reliability of poor-channel condition users through employing a dedicated relay/user. Here, a relay/user can adopt one or two operation modes relying on the listening and forwarding phases with half-duplex (HD) relaying or full-duplex (FD) relaying. In HD relay, the SE of the cooperative system suffers in comparison with direct transmission, since it requires two time-orthogonal phases to carry out reception and re-transmission of the information. Meanwhile, FD relay avoids the SE loss in HD relay through simultaneous listening/forwarding signals in the same frequency band. However, one of the main drawbacks of FD relay is the existence of residual self-interference (SI) at the relay’s receiver, which dramatically degrades the system performance. In [19], Tung  et al. proposed a novel cooperative direct and relay transmission for a NOMA-based IoT relay network, where one master IoT node simultaneously serves a cell-edge user and IoT user acting as a decode-and-forward (DF) relay in HD mode, aiming to enhance the ergodic sum capacity. According to [19], the combination of a cellular NOMA system with an IoT network opens a new direction for further improving the SE and performance of cell-edge users. Likewise, Rauniyar  et al. studied the performance of a wireless powered cooperative NOMA-based IoT relay system with the presence of an interfering signal in [20]. The authors in [21] studied a wireless powered cooperative NOMA system where the relay operates with a hybrid protocol (i.e., the DF protocol will perform if the relay successfully decodes the received signals; conversely, the amplify-and-forward (AF) protocol will be adopted) in an untrusted relay scenario. Recently, some works have investigated the performance of FD cooperative NOMA systems [22,23,24,25,26,27,28,29]. In [22], Zhang  et al. proposed an FD device-to-device-aided cooperative NOMA system to improve the outage performance of the NOMA-weak user. Toward practical deployment, Xu  et al. introduced a novel network-coded multiple access for an FD cooperative NOMA system [23], under which physical-layer network coding is employed at the NOMA user to demodulate signals with higher probability. However, the EH technique was not regarded in these works.

A combination of FD, SWIPT, and NOMA to improve the spectral usage and energy efficiency for wireless networks has been investigated. The OP and ergodic rates of a wireless powered SWIPT NOMA system were analyzed in [24]. The impacts of three SI scenarios on FD SWIPT NOMA with beam forming have been investigated in [25]: SI fully removed, utilizing SI to provide extra energy during EH process, and imperfect SI. The impact of multi-antennas cognitive relay for an FD NOMA system with non-linear EH was investigated in [26]. Minimizing the transmit power requirement for full and partial CSIT of FD SWIPT NOMA systems was investigated in [27]. Unlike the works of [24,25,26,27], Agrawal  et al. investigated the performance of SWIPT NOMA FD relay networks in [28], where the performance of a far away user can be improved significantly through the assistance of a battery at the relay. An adaptive power allocation scheme for a SWIPT-enabled FD cooperative NOMA system was studied in [29]. Recently, the authors in [30] investigated outage and throughput cooperative full-duplex relaying based NOMA with EH. Most of these mentioned studies extensively explored the impact of deploying SWIPT-enabled FD NOMA networks. The direct links between a source node and a cell-edge user and relaying links have not been considered (except [30]). Additionally, the Nakagami-*m* distribution is known to perform better than a Rayleigh fading distribution for modeling channels in a NOMA relay system [31]. Nevertheless, it did not receive attention in [19,20,21,22,23,24,25,26,27,28,29,30].

Yet, to the best of our knowledge, the research on full-duplex SWIPT CNOMA-based IoT is still an open question. Furthermore, the impact of iSIC for practical NOMA networks is still a challenging question and needs to be investigated. Motivated by the above observations, we propose an FD SWIPT cooperative NOMA-based IoT relay network over Nakagami-*m* fading channels where two scenarios with and without a direct link between the source node and cell-edge user are considered (in [32], the authors proposed a relay selection scheme to evaluate the outage probability and average achievable rate of a multi-user system without EH. A machine learning solution to improve harvesting energy based on clustering users was proposed in [10]. However, relay selection and clustering user issues are not focused on in this paper and will be left in the next works). We also propose a low-complexity algorithm to maximize the system throughput by optimizing the time-switching (TS) factor. The main contributions of this paper can be summarized as follows:

- First, we propose an FD SWIPT cooperative NOMA-based IoT relay system with pSIC and iSIC, where one master IoT node acts as an FD DF relay to enhance a cell-edge user’s performance. Specifically, to help a source node simultaneously communicate with a cell-edge user, the relay performs pairing of the received signal of a cell-edge user with an IoT user via the NOMA protocol. At the cell-edge user, a selection combining (SC) (the SC technique has been widely used for cell-edge users in the literature for improving wireless system performance. This is because it has the lowest implementation compared to maximal ratio combing (MRC) and equal-gain combining (EGC), which are required for full knowledge of the channel state information [33,34]) technique is employed to improve performance. We also consider two scenarios with a direct and a non-direct link between the source node and cell-edge user.
- Secondly, we analyze the performance analysis of the proposed system in terms of the OP, system throughput, EE, and ergodic capacity. Exact closed-form analytical expressions and approximate expressions for the OP, system throughput, EE, and ergodic capacity are derived accordingly. To reveal useful insights into the proposed system, the asymptotic expression for the system throughput is also given.
- Thirdly, we propose a low complexity algorithm to find the optimal TS factor that guarantees maximal system throughput. By performing our proposed algorithm, the system throughput can be vastly improved.
- Finally, we show through numerical results that our proposed system always outperforms its orthogonal multiple access (OMA) counterpart in terms of the OP, system throughput, and ergodic sum capacity under pSIC and achieves better performance for a low to medium signal-to-noise ratio (SNR). Furthermore, the system performance of the cell-edge user is significantly enhanced in the direct link scenario using SC compared to the non-direct link scenario when the residual SI caused by the iSIC process increases to larger than 40%.

**Notation:** We use $\exp\left(\cdot \right)$ to denote the exponential function, $k!=k\times \left(k-1\right)\times \cdots \times 1$, $\mathrm{Pr}\left(\cdot \right)$ represents the probability, $\mathrm{CN}(0,\sigma^{2})$ represents a zero-mean complex Gaussian distribution with variance $\sigma^{2}$, and $E\left\{\cdot \right\}$ denotes the expectation operation. Finally, $f_{X}\left(\cdot \right)$ and $F_{X}\left(\cdot \right)$ denote the probability density function (PDF) and cumulative distribution function (CDF) of a random variable *X*, respectively.

## 2. System Model

**Figure 1:**
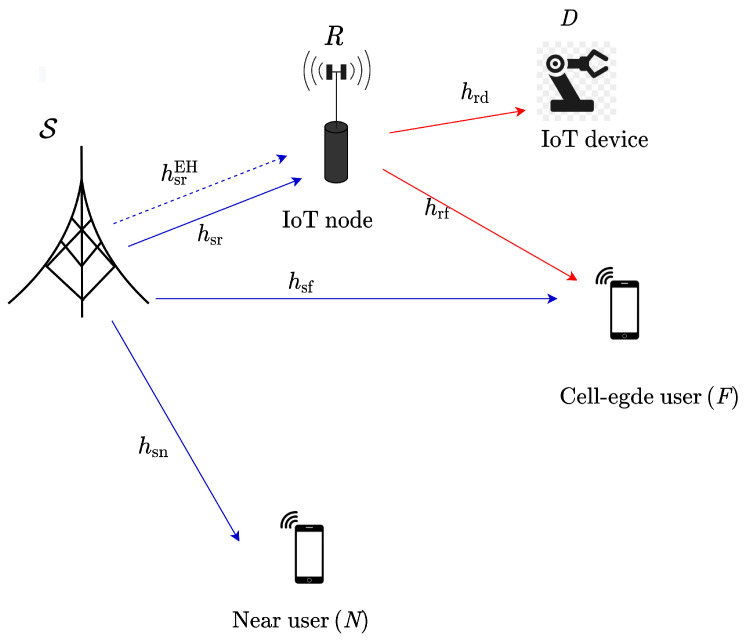
An illustration of full-duplex SWIPT NOMA-based IoT relay networks.

As shown in Figure 1, we consider a wireless-powered full-duplex cooperative NOMA-based IoT relay system, where a source node (S) broadcasts its information to two users (i.e., a cell-center user called N and a cell-edge user called F) through the assistance of one master IoT node serving one IoT user (the master IoT node has a role as a source of the IoT network, while the IoT user can be a wireless camera or sensor [19,20,35,36]) (named D), acting as DF relay (i.e., R). Since the master IoT node is an energy-limited device, it has to harvest energy from the RF signals radiating from S. We assume that all nodes are equipped with single antennas while R is is equipped with two separate antennas, one for transmission and the other for reception. We also assume that perfect knowledge of the channel state information is available at each terminal [37].

**Figure 2:**
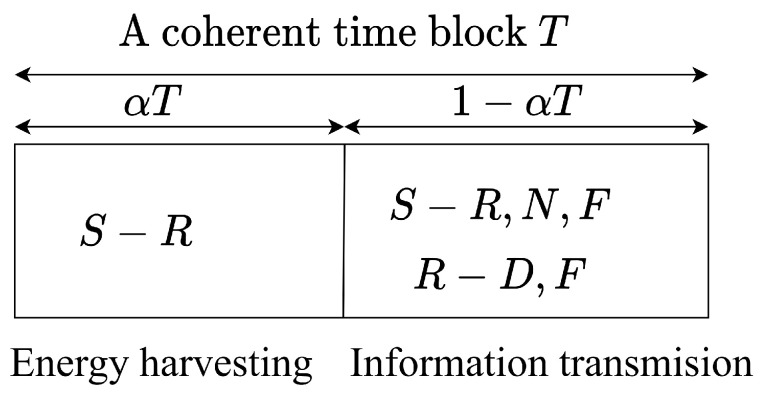
Energy harvesting phase and transmission phase of the TS protocol.

The entire communication process is divided into two consecutive phases consisting of EH and information transmission, as depicted in Figure 2. During the first period of αT, R harvests energy from S and works in HD mode. In the next period of $\left(1-\alpha \right)T$, S transmits the superimposed signals to R as well as the users, where R receives the signal from S, re-encodes, and then transmits a superimposed signal to the destinations simultaneously in FD mode.

### 2.1. Channel Model

We denote $h_{XY}$ by the channel coefficients between any two nodes in the network, where X∈{s,r}, Y∈{r,n,f,d}. It is assumed that wireless links are independent and identically distributed (i.i.d), modeled as Nakagami-*m* fading channels with fading parameter $m_{XY}$ being an integer, and $E\left\{{\left|h_{XY}\right|}^{2}\right\}=\Omega_{XY}$. The channel power gains ${\left|h_{XY}\right|}^{2}$ is subjected to Gamma-distributed random variables with parameter $\Omega_{XY}=L{\left(\frac{d_{XY}}{d_{0}}\right)}^{-\theta }$, where $d_{XY}$ is the normalized distance between node *X* and node *Y*, $d_{0}$ denotes the reference distance, θ is the path-loss exponent, and L is the average signal power attenuation at $d_{0}$. Accordingly, the CDF and PDF of ${\left|h_{XY}\right|}^{2}$ are given as follows [37]:

(1) $$\begin{array}{cc} \; & F_{{\left|h_{XY}\right|}^{2}}\left(x\right)=1-\exp\left(-\frac{xm_{XY}}{\Omega_{XY}}\right)\sum_{k=0}^{m_{XY}-1}\frac{1}{k!}{\left(\frac{xm_{XY}}{\Omega_{XY}}\right)}^{k}, \end{array}$$

(2) $$\begin{array}{cc} \; & f_{{\left|h_{XY}\right|}^{2}}\left(x\right)=\frac{x^{m_{XY}-1}}{\left(m_{XY}-1\right)!}{\left(\frac{m_{XY}}{\Omega_{XY}}\right)}^{m_{XY}}\exp\left(-\frac{m_{XY}x}{\Omega_{XY}}\right). \end{array}$$

Owing to the advancement of SI cancellation techniques, the FD relay can attain about 80–110 dB of SI suppression [38]; thus, some recent works have ignored the importance of SI such as in [22,23] where the authors considered the mount of residual SI as constant. Meanwhile, the impact of the SI channel random variable was well studied with Rayleigh quasi-static fading in [26] and investigated with Nakagami-*m* fading channel in [39]. Since the distance between the transmitter and receiver is too small and cannot be modeled with the path-loss scenario, we consider the parameter $\Omega_{\mathrm{rr}}$ corresponding the channel power gain ${\left|h_{\mathrm{rr}}\right|}^{2}$ from −20 to −5 dB, which is similarly assumed in [28] but with a lower range i.e., −40 to −20 dB.

### 2.2. Energy Harvesting (EH) and Data Transmission Processes

In the EH phase, by applying the TS mechanism, R simultaneously collects energy from S during the prior period time of αT, where *T* and α present the coherent block time and TS factor, respectively. The harvested energy at the input of the EH circuits of R can be expressed as

(3) $$\begin{array}{c} E_{R}=\mu \alpha TP_{S}{\left|h_{\mathrm{sr}}^{\mathrm{EH}}\right|}^{2}, \end{array}$$

where $\mu \in \left(0,1\right)$ denotes the energy conversion efficiency and $P_{S}$ is the transmit power of R. All the energy harvested during the EH phase is consumed at R while forwarding the decoded signal to the destination users. The transmit power of R can be obtained from the harvested energy *E* in (3) as

(4) $$\begin{array}{c} P_{R}=\frac{E_{R}}{\left(1-\alpha \right)T}=\omega P_{S}{\left|h_{\mathrm{sr}}^{\mathrm{EH}}\right|}^{2},\omega =\frac{\mu \alpha }{\left(1-\alpha \right)}. \end{array}$$

In the information transmission phase, S broadcasts the superimposed signal $x_{}=\sqrt{P_{S}\delta_{1}}x_{F}$$+\sqrt{P_{S}\left(1-\delta_{1}\right)}x_{N}$ to the users and R based on the NOMA protocol, where $x_{N}$ and $x_{F}$ represent the signals of N and F, respectively, i.e., $E\left\{{\left|x_{N}\right|}^{2}\right\}=E\left\{{\left|x_{F}\right|}^{2}\right\}=1$, and $\delta_{1}$ is the power allocation (PA) factor. We assume that the channel ${\left|h_{\mathrm{sf}}\right|}^{2}$ is less than channel ${\left|h_{\mathrm{sn}}\right|}^{2}$; thus, more power is allocated for F, yielding $\delta_{1}\in \left(0.5,1\right)$. The observation signals at R, N, and F transmitted by S can be respectively expressed as follows:

(5) $$\begin{array}{cc} \; & y_{\mathrm{sn}}=\left(\sqrt{\left(1-\delta_{1}\right)P_{S}}x_{N}+\sqrt{\delta_{1}P_{S}}x_{F}\right)h_{\mathrm{sn}}+n_{\mathrm{sn}}, \end{array}$$

(6) $$\begin{array}{cc} \; & y_{\mathrm{sf}}=\left(\sqrt{\left(1-\delta_{1}\right)P_{S}}x_{N}+\sqrt{\delta_{1}P_{S}}x_{F}\right)h_{\mathrm{sf}}+n_{\mathrm{sf}}, \end{array}$$

(7) $$\begin{array}{cc} \; & y_{\mathrm{sr}}=\left(\sqrt{\left(1-\delta_{1}\right)P_{S}}x_{N}+\sqrt{\delta_{1}P_{S}}x_{F}\right)h_{\mathrm{sr}}+x_{R}h_{\mathrm{rr}}+n_{\mathrm{sr}}, \end{array}$$

where $n_{\mathrm{sr}}∼\mathrm{CN}\left(0,\sigma^{2}\right)$, $n_{\mathrm{sf}}∼\mathrm{CN}\left(0,\sigma^{2}\right)$, and $n_{\mathrm{sf}}∼\mathrm{CN}\left(0,\sigma^{2}\right)$ are the adaptive white Gaussian noise (AWGN) at N, F, and R, respectively. The respectively instantaneous signal-to-plus-noise ratios (SINRs) at N for decoding signals $x_{f}$ and $x_{N}$ by using successive interference cancellation (SIC) can be respectively given as

(8) $$\begin{array}{cc} \; & \gamma_{\mathrm{sn}}^{x_{F}}=\frac{\delta_{1}P_{S}{\left|h_{\mathrm{sn}}\right|}^{2}}{P_{S}\left(1-\delta_{1}\right){\left|h_{\mathrm{sn}}\right|}^{2}+\sigma^{2}}, \end{array}$$

(9) $$\begin{array}{cc} \; & \gamma_{\mathrm{sn}}^{x_{n}}=\frac{\left(1-\delta_{1}\right)P_{S}{\left|h_{\mathrm{sn}}\right|}^{2}}{\psi_{1}\delta_{1}P_{S}{\left|h_{\mathrm{sn}}\right|}^{2}+\sigma^{2}}, \end{array}$$

where $\psi_{1}$ is the level of residual SI caused by the imperfect SIC process. The received SINRs for decoding signal $x_{F}$ at R and F can be respectively expressed as

(10) $$\begin{array}{cc} \; & \gamma_{\mathrm{sr}}^{x_{F}}=\frac{\delta_{1}P_{S}{\left|h_{\mathrm{sr}}\right|}^{2}}{P_{S}\left(1-\delta_{1}\right){\left|h_{\mathrm{sr}}\right|}^{2}+P_{R}{\left|h_{\mathrm{rr}}\right|}^{2}+\sigma^{2}}, \end{array}$$

(11) $$\begin{array}{cc} \; & \gamma_{\mathrm{sf}}^{x_{F}}=\frac{\delta_{1}P_{S}{\left|h_{\mathrm{sf}}\right|}^{2}}{P_{S}\left(1-\delta_{1}\right){\left|h_{\mathrm{sf}}\right|}^{2}+\sigma^{2}}. \end{array}$$

At R, after decoding the signal of F, R re-encodes and broadcasts a superimposed signal $x_{R}=\sqrt{P_{R}\left(1-\delta_{2}\right)}x_{F}+\sqrt{P_{R}\delta_{2}}x_{d}$ to F and D, where $x_{d}$ presents the signal of D, i.e., $E\left\{{\left|x_{d}\right|}^{2}\right\}=1$, and $\delta_{2}$ is the PA factor. To satisfy the QoS requirement of the IoT zone [19], more power is devoted to D and less power is devoted to F, yielding $\delta_{2}\in \left(0.5,1\right)$. The observation signal at F and D transmitted by R can be respectively expressed as

(12) $$\begin{array}{cc} \; & y_{\mathrm{rf}}=\left(\sqrt{P_{S}\left(1-\delta_{1}\right)}x_{N}+\sqrt{P_{S}\delta_{1}}x_{F}\right)h_{\mathrm{rf}}+n_{\mathrm{rf}}, \end{array}$$

(13) $$\begin{array}{cc} \; & y_{\mathrm{rd}}=\left(\sqrt{P_{S}\left(1-\delta_{1}\right)}x_{N}+\sqrt{P_{S}\delta_{1}}x_{F}\right)h_{\mathrm{rd}}+n_{\mathrm{rd}}, \end{array}$$

where $n_{\mathrm{rf}}∼\mathrm{CN}\left(0,\sigma^{2}\right)$ and $n_{\mathrm{rd}}∼\mathrm{CN}\left(0,\sigma^{2}\right)$ are the AWGN at F, and D, respectively. By following the NOMA principle, F first decodes signal $x_{D}$ and then subtracts this signal before decoding its own signal. The received SINR at F to decode $x_{D}$ and $x_{F}$ can be respectively computed as

(14) $$\begin{array}{cc} \; & \gamma_{\mathrm{rf}}^{x_{D}}=\frac{\delta_{2}P_{R}{\left|h_{\mathrm{rf}}\right|}^{2}}{P_{R}\left(1-\delta_{2}\right){\left|h_{\mathrm{rf}}\right|}^{2}+\sigma^{2}}, \end{array}$$

(15) $$\begin{array}{cc} \; & \gamma_{\mathrm{rf}}^{x_{F}}=\frac{\left(1-\delta_{2}\right)P_{R}{\left|h_{\mathrm{rf}}\right|}^{2}}{\psi_{2}\delta_{2}P_{R}{\left|h_{\mathrm{rf}}\right|}^{2}+\sigma^{2}}, \end{array}$$

where $\psi_{2}$ is the residual SI coefficient. Meanwhile, D directly decodes signal $x_{D}$ with the received SINR as

(16) $$\begin{array}{c} \gamma_{\mathrm{rd}}^{x_{D}}=\frac{\delta_{2}P_{R}{\left|h_{\mathrm{rd}}\right|}^{2}}{P_{R}\left(1-\delta_{2}\right){\left|h_{\mathrm{rd}}\right|}^{2}+\sigma^{2}}. \end{array}$$

For the DF protocol, the achievable rate of the considered network is calculated based solely on the weakest hop. Therefore, the end-to-end (e2e) achievable rate of D is acquired from (14), and (16) as [19]:

(17) $$\begin{array}{c} C_{D}=\left(1-\alpha \right)\log_{2}\left(1+\min\left\{\gamma_{\mathrm{rf}}^{x_{D}},\gamma_{\mathrm{rd}}^{x_{D}}\right\}\right), \end{array}$$

and the achievable rate of N is acquired from (9) as

(18) $$\begin{array}{c} C_{N}=\left(1-\alpha \right)\log_{2}\left(1+\gamma_{\mathrm{sn}}^{x_{N}}\right). \end{array}$$

We consider two scenarios based on the existence of direct links between S and F because of the deep shadowing phenomenon: (i) with a direct link (DL) and (ii) a non-direct link (NL). If the DL link does not exist, the e2e achievable rate of F is acquired from (8), (10) and (15) as:

(19) $$\begin{array}{c} C_{F}^{\mathrm{NL}}=\left(1-\alpha \right)\log_{2}\left(1+\min\left\{\gamma_{\mathrm{sn}}^{x_{F}},\gamma_{\mathrm{sr}}^{x_{F}},\gamma_{\mathrm{rf}}^{x_{F}}\right\}\right). \end{array}$$

If the DL link exists, F needs to combine two signals from S and R by employing the SC technique. Thus, from (8), (10), (11) and (15), the e2e achievable rate of F is acquired as:

(20) $$\begin{array}{c} C_{F}^{\mathrm{DL}}\;=\;\left(1-\alpha \right)\log_{2}\left(1+\min\left\{\gamma_{\mathrm{sn}}^{x_{F}},\gamma_{\mathrm{sr}}^{x_{F}},\max\left\{\gamma_{\mathrm{sf}}^{x_{F}},\gamma_{\mathrm{rf}}^{x_{F}}\right\}\right\}\right). \end{array}$$

For comparison purposes, we consider full time-division multiple access, which is representative of OMA as the benchmark schemes. The whole information transmission phase is performed with four consecutive time slots, where each signal is transmitted in a separate time slot. In particular, the achieved power of R is split equally for their transmission (i.e., with F and D). In this case, the e2e achievable rates of F, N, and D are calculated respectively as follows:

(21) $$\begin{array}{cc} \; & C_{F}^{\mathrm{OMA}}=\frac{\left(1-\alpha \right)}{4}\log_{2}\left(1+\min\left\{\frac{P_{S}{\left|h_{sr}\right|}^{2}}{\sigma_{R}^{2}},\frac{0.5P_{R}{\left|h_{\mathrm{rf}}\right|}^{2}}{\sigma_{F}^{2}}\right\}\right), \end{array}$$

(22) $$\begin{array}{cc} \; & C_{N}^{\mathrm{OMA}}=\frac{\left(1-\alpha \right)}{4}\log_{2}\left(1+\frac{P_{S}{\left|h_{\mathrm{sn}}\right|}^{2}}{\sigma_{N}^{2}}\right), \end{array}$$

(23) $$\begin{array}{cc} \; & C_{D}^{\mathrm{OMA}}=\frac{\left(1-\alpha \right)}{4}\log_{2}\left(1+\frac{0.5P_{R}{\left|h_{\mathrm{rd}}\right|}^{2}}{\sigma_{D}^{2}}\right). \end{array}$$

## 3. Performance Analysis

### 3.1. Outage Probability (OP)

The outage event can be defined as the probability that the achievable rate of a receiver is lower than a specific target rate. We denote $R_{N}$, $R_{D}$, and $R_{F}$ as the constant rates of N, D, and F, respectively. Let $\gamma_{N}=2^{\frac{R_{N}}{1-\alpha }}-1$, $\gamma_{D}=2^{\frac{R_{D}}{1-\alpha }}-1$, and $\gamma_{F}=2^{\frac{R_{F}}{1-\alpha }}-1$ be the SINR threshold for decoding the signals $x_{D}$, $x_{N}$, and $x_{F}$, respectively. In what follows, we derive OP expressions for D, N, and F.

#### 3.1.1. OP of Cell-Center User

The outage event of N occurs when the achievable rate is lower the given target data rate, $R_{N}$. Henceforth, the OP of N can be expressed as

(24) $$\begin{array}{cc} P_{\mathrm{out}}^{N} & =\mathrm{Pr}\left[\left(1-\alpha \right)\log_{2}\left(1+\gamma_{\mathrm{sn}}^{x_{N}}\right)\leq R_{N}\right]=\mathrm{Pr}\left[\frac{\left(1-\delta_{1}\right)P_{S}{\left|h_{\mathrm{sn}}\right|}^{2}}{\psi_{1}\delta_{1}P_{S}{\left|h_{\mathrm{sn}}\right|}^{2}+\sigma^{2}}\leq \gamma_{N}\right]. \end{array}$$

From (24), by setting $\beta_{1}=\frac{1-\delta_{1}}{\delta_{1}}$, $\rho =\frac{P_{S}}{\sigma^{2}}$, $V_{1}=\frac{m_{\mathrm{sn}}}{\delta_{1}\rho \Omega_{\mathrm{sn}}}$ and using the CDF function in (1), after some simple manipulation steps, we obtained the OP of N in the following theorem.

**Theorem** **1.** *The exact closed-form expression for OP of N can be derived as*

(25) $$\begin{array}{c} P_{\mathrm{out}}^{N}=1-\exp\left(-\frac{\gamma_{N}V_{1}}{\beta_{1}-\gamma_{N}\psi_{1}}\right)\sum_{k_{\mathrm{sn}}=0}^{m_{\mathrm{sn}}-1}\frac{1}{k_{\mathrm{sn}}!}{\left(\frac{\gamma_{N}V_{1}}{\beta_{1}-\gamma_{N}\psi_{1}}\right)}^{k_{\mathrm{sn}}}, \end{array}$$

*for $\beta_{1}-\gamma_{N}\psi_{1}>0$ and $P_{\mathrm{out}}^{N}=1$ for $\beta_{1}-\gamma_{N}\psi_{1}\leq 0$.*

#### 3.1.2. OP of IoT User

The outage event of D occurs when the e2e achievable rate is lower than the given target data rate, $R_{D}$. The OP of D can be attained as

(26) $$\begin{array}{c} P_{\mathrm{out}}^{D}=\mathrm{Pr}\left[\left(1-\alpha \right)\log_{2}\left(1+\min\left\{\gamma_{\mathrm{rf}}^{x_{D}},\gamma_{\mathrm{rd}}^{x_{D}}\right\}\right)\leq R_{D}\right]. \end{array}$$

**Theorem** **2.** *The exact analytical closed-form expression for OP of D can be attained as*

(27) $$\begin{array}{cc} P_{\mathrm{out}}^{D}=1 & -2\sum_{k_{\mathrm{rf}}=0}^{m_{\mathrm{rf}}-1}\sum_{k_{\mathrm{rd}}=0}^{m_{\mathrm{rd}}-1}\frac{A^{k_{\mathrm{rf}}}B^{k_{\mathrm{rd}}}{\left(A+B\right)}^{\frac{m_{\mathrm{sr}}-l}{2}}}{k_{\mathrm{rf}}!k_{\mathrm{rd}}!\left(m_{\mathrm{sr}}-1\right)!}{\left(\frac{\gamma_{D}V_{2}}{\beta_{2}-\gamma_{D}}\right)}^{\frac{m_{\mathrm{sr}}+l}{2}}K_{m_{\mathrm{sr}}-l}\left(2\sqrt{\frac{\gamma_{D}V_{2}\left(A+B\right)}{\beta_{2}-\gamma_{D}}}\right), \end{array}$$

*where $l=k_{\mathrm{rf}}+k_{\mathrm{rd}}$ and $K_{l}\left(\cdot \right)$ is the lth-order modified Bessel function of the second kind, which is developed as a standard function in some popular mathematical software packages (i.e., Matlab, Maple, and Mathematica) for $\beta_{2}>\gamma_{D}$, and otherwise $P_{\mathrm{out}}^{D}=1$.*

**Proof.**  See Appendix A.   □

#### 3.1.3. OP of Cell-Edge User

The outage event of F occurs when the e2e achievable rate is lower than the given target data rate, $R_{F}$. The OP of F in case of NL can be expressed as

(28) $$\begin{array}{c} P_{\mathrm{out}}^{F,\mathrm{NL}}=\mathrm{Pr}\left[\min\left\{\gamma_{\mathrm{sn}}^{x_{F}},\gamma_{\mathrm{sr}}^{x_{F}},\gamma_{\mathrm{rf}}^{x_{F}}\right\}\leq \gamma_{F}\right]. \end{array}$$

**Theorem** **3.** *The exact closed-form analytical expression for OP of F in the case of NL can be attained as*

(29) PoutF,NL=1−exp−γF1−δ1−1msn1/β1−γFρΩsn−ΔmsrΩsrρ∑ksn=0msn−11ksn!γF1−δ1−1msn1/β1−γFρΩsnksn[∑ksr=0msr−1∑n=0ksrksrnmsrΩsrωρksr−nmrrΩrrmsrΓmsr+nΓmrr+nΓmsrΓmrrΔωmsr+n−ksrUmsr+n,msr−mrr+1,mrrΔωΩrr]∑krf=0mrf−12V3Amsr+krf2krf!msr−1!Kmsr−krf2V3A.

**Proof.**  See Appendix B.   □

In the case of DL between S and F, the OP of F can be expressed as

(30) $$\begin{array}{c} P_{\mathrm{out}}^{F,\mathrm{DL}}=\mathrm{Pr}\left[\min\left\{\gamma_{\mathrm{sn}}^{x_{F}},\gamma_{\mathrm{sr}}^{x_{F}},\max\left\{\gamma_{\mathrm{sf}}^{x_{F}},\gamma_{\mathrm{rf}}^{x_{F}}\right\}\right\}\leq \gamma_{F}\right]. \end{array}$$

**Theorem** **4.** *The exact closed-form analytical expression for OP of F in the case of DL can be expressed as follows: If $\gamma_{F}>\frac{\delta_{1}}{1-\delta_{1}}$, $P_{\mathrm{out}}^{F,\mathrm{DL}}=1$. Otherwise, if $\gamma_{F}<\frac{1}{\psi_{2}\beta_{2}}$, the OP of F can be obtained as*

(31) PoutF,DL,C1=1−exp−γF1−δ1−1msn1/β1−γFρΩsn−ΔmsrΩsrρ∑ksn=0msn−11ksn!γF1−δ1−1msn1/β1−γFρΩsnksn×∑ksr=0msr−1∑n=0ksrksrnmsrΩsrωρksr−nmrrΩrrmsrΓmsr+nΓmrr+nΓmsrΓmrrΔωmsr+n−ksrUmsr+n,msr−mrr+1,mrrΔωΩrr[∑krf=0mrf−12V3Amsr+krf2krf!msr−1!Kmsr−krf2V3A+exp−ΔmsfρΩsf∑ksf=0msf−1ΔmsfρΩsfksfksf!1−∑krf=0mrf−12V3Amsr+krf2krf!msr−1!Kmsr−krf2V3A],

*else the OP of F is given as*

(32) PoutF,DL,C2=1−exp−γF1−δ1−1msn1/β1−γFρΩsn−ΔmsfρΩsf∑ksn=0msn−11ksn!γF1−δ1−1msn1/β1−γFρΩsnksn×exp−ΔmsrΩsrρ∑ksf=0msf−1ΔmsfρΩsfksfksf!∑ksr=0msr−1∑n=0ksrksrnmsrΩsrωρksr−nmrrΩrrmsr×Γmsr+nΓmrr+nΓmsrΓmrrΔωmsr+n−ksrUmsr+n,msr−mrr+1,mrrΔωΩrr.

**Proof.**  See Appendix B.   □

### 3.2. System Throughput

In this subsection, we derive and evaluate the corresponding system throughput of two schemes, with and without direct links between S and F. The system throughput is defined as the total amount of information, which is transmitted per unit time with a constant rate, *R*, relying on the performance of OPs due to the wireless fading channels. Thenceforth, the system throughput in delay-limited transmission mode depending on the TS mechanism can be expressed as

(33) $$\begin{array}{c} T_{\mathrm{put}}^{G}\;=\;R_{N}\left(1-P_{\mathrm{out}}^{N}\right)+R_{D}\left(1-P_{\mathrm{out}}^{D}\right)+R_{F}\left(1-P_{\mathrm{out}}^{F,G}\right), \end{array}$$

where G∈{NL,DL}. From (33), we can straightforwardly achieve the upper bound and lower bound of the system throughput as

(34) $$\begin{array}{c} 0\leq T_{\mathrm{put}}^{G}\leq R_{N}+R_{D}+R_{F}. \end{array}$$

### 3.3. Average Energy Efficiency (EE)

The average EE is defined as the ratio of the achievable data rate to the total power consummation for the whole system. From this definition, we determined that the average EE of the considered system setup can be expressed as

(35) $$\begin{array}{c} \mu_{\mathrm{EE}}^{G}=\frac{T_{\mathrm{put}}^{G}}{P_{S}+P_{R}+P_{c}}, \end{array}$$

where $P_{c}$ presents the total static power consumed by the circuit during the EH of S and information transmission of S and R.

### 3.4. Ergodic Sum Capacity (ESC)

Ergodic capacity (EC) is defined as the user’s target data rates adjusted with respect to their channel condition, which can be expressed as

(36) $$\begin{array}{cc} E\left\{C\right\} & =\left(1-\alpha \right)\int_{0}^{\infty }\log_{2}\left(1+x\right)f_{X}\left(x\right)dx=\left(1-\alpha \right)\int_{0}^{\infty }\frac{1-F_{X}\left(x\right)}{\ln2\left(1+x\right)}dx. \end{array}$$

From this definition, the ESC of the proposed system scheme can be calculated as ${\bar{C}}_{\mathrm{sum}}^{G}={\bar{C}}_{N}+{\bar{C}}_{D}+{\bar{C}}_{F}^{G}$. Based on the derivation OP (i.e., CDF) of N,D, and F, the considered system setup can operate without outage if and only if these conditions (these conditions can be found based on the results analysis of Theorem 1 and the proof of other theorems in Appendix A and Appendix B) are satisfied as

(37) $$\begin{array}{c} \left\{\begin{array}{cc} \gamma_{D}<\frac{\delta_{2}}{1-\delta_{2}}, & \;\mathrm{for}\;D,\\ \psi_{1}\delta_{1}\gamma_{N}<1-\delta_{1}, & \;\mathrm{for}\;N,\\ \left(\gamma_{F}<\frac{\delta_{1}}{\left(1-\delta_{1}\right)}\right)⋂\left(\psi_{2}\delta_{2}\gamma_{F}<1-\delta_{2}\right), & \mathrm{for}\;F\;\mathrm{with}\;\mathrm{NL},\\ \gamma_{F}\in \left\{C_{1},C_{2}\right\}, & \;\mathrm{for}\;F\;\mathrm{with}\;\mathrm{DL}. \end{array}\right. \end{array}$$

#### 3.4.1. EC of Cell-Center User

The EC of N can be expressed as

(38) $$\begin{array}{cc} \; & {\bar{C}}_{N}=\frac{1-\alpha }{\ln2}\left\{\begin{array}{cc} \int_{0}^{\infty }\frac{1-F_{\gamma_{N}}\left(x\right)}{\left(1+x\right)}dx, & \mathrm{for}\;\mathrm{pSIC},\;i.e.,\;\psi_{1}=0,\\ \int_{0}^{\frac{1-\delta_{1}}{\psi_{1}\delta_{1}}}\frac{1-F_{\gamma_{N}}\left(x\right)}{\left(1+x\right)}dx, & \mathrm{for}\;\mathrm{iSIC},\;i.e.,\;\psi_{1}\in \left(0,1\right). \end{array}\right. \end{array}$$

Accordingly, the EC of N in the cases of pSIC and iSIC can be expressed in the following propositions.

**Proposition** **1.** *The exact closed-form analytical expression for the EC of N with pSIC can be attained as*

(39) $$\begin{array}{cc} {\bar{C}}_{N}^{\mathrm{pSIC}}= & \frac{1-\alpha }{\ln2}\sum_{k_{\mathrm{sn}}=0}^{m_{\mathrm{sn}}-1}\frac{1}{k_{\mathrm{sn}}!}{\left(\frac{V_{1}}{\beta_{1}}\right)}^{k_{\mathrm{sn}}}\left[{\left(\;-\;1\right)}^{k_{\mathrm{sn}}-1}\exp\left(\frac{V_{1}}{\beta_{1}}\right)\mathrm{Ei}\left(-\frac{V_{1}}{\beta_{1}}\right)+\sum_{n=1}^{k_{\mathrm{sn}}}\frac{\Gamma \left(n\right){\left(-1\right)}^{n-k_{\mathrm{sn}}}}{{\left(V_{1}/\beta_{1}\right)}^{n}}\right], \end{array}$$

*where Ei(·) is the exponential integral function in Equation (8.211.1) of [40].*

**Proposition** **2.** *The closed-form analytical approximate expression for EC of N with iSIC can be attained as*

(40) $$\begin{array}{c} {\bar{C}}_{N}^{\mathrm{iSIC}}\;=\;\frac{1-\alpha }{\ln2}\sum_{k_{\mathrm{sn}}=0}^{m_{\mathrm{sn}}-1}\frac{\Xi_{1}}{k_{\mathrm{sn}}!}\sum_{i=1}^{N}\frac{\pi \sqrt{1-w_{i}^{2}}}{N\left(1+{\bar{\phi }}_{1}^{i}\right)}\exp\left(-\frac{{\bar{\phi }}_{1}^{i}V_{1}}{\beta_{1}-{\bar{\phi }}_{1}^{i}\psi_{1}}\right){\left(\frac{{\bar{\phi }}_{1}^{i}V_{1}}{\beta_{1}-{\bar{\phi }}_{1}^{i}\psi_{1}}\right)}^{k_{\mathrm{sn}}}, \end{array}$$

*where $\Xi_{1}=\frac{\beta_{1}}{2\psi_{1}}$ and ${\bar{\phi }}_{1}^{i}=\Xi_{1}\left(w_{i}+1\right)$.*

**Proof.**  See Appendix C.   □

#### 3.4.2. EC of IoT User

The EC of D can be expressed as

(41) $$\begin{array}{c} {\bar{C}}_{D}=\frac{1-\alpha }{\ln2}\int_{0}^{\beta_{2}}\frac{1-F_{\gamma_{D}}\left(x\right)}{\left(1+x\right)}dx. \end{array}$$

Unfortunately, the exact closed-form expression of (41) for D cannot be derived after plugging in the CDF of $\gamma_{D}$ since the CDF contains the multi-summation terms of Bessel function. However, it can be solved using numerical solutions, such as Gaussian–Chebyshev quadrature (GCQ), to compute a tightly bounded expression for the EC of D with low complexity and high accuracy. The closed-form approximated can be expressed in the following proposition.

**Proposition** **3.** *The closed-form analytical approximate expression for EC of D can be attained as*

(42) $$\begin{array}{cc} {\bar{C}}_{D}= & \frac{2\Xi_{2}\left(1-\alpha \right)}{\ln2}\sum_{k_{\mathrm{rf}}=0}^{m_{\mathrm{rf}}-1}\sum_{k_{\mathrm{rd}}=0}^{m_{\mathrm{rd}}-1}\sum_{i=1}^{N}\frac{\pi \sqrt{1-w_{i}^{2}}A^{k_{\mathrm{rf}}}B^{k_{\mathrm{rd}}}{\left(A+B\right)}^{\frac{m_{\mathrm{sr}}-l}{2}}}{Nk_{\mathrm{rf}}!k_{\mathrm{rd}}!\left(m_{\mathrm{sr}}-1\right)!\left(1+{\bar{\phi }}_{2}^{i}\right)}{\left(\frac{{\bar{\phi }}_{2}^{i}V_{2}}{\beta_{2}-{\bar{\phi }}_{2}^{i}}\right)}^{\frac{m_{\mathrm{sr}}+l}{2}}\\ \; & \times K_{m_{\mathrm{sr}}-l}\left(2\sqrt{\frac{{\bar{\phi }}_{2}^{i}V_{2}\left(A+B\right)}{\beta_{2}-{\bar{\phi }}_{2}^{i}}}\right), \end{array}$$

*where $\Xi_{2}=\beta_{2}/2$ and ${\bar{\phi }}_{2}^{i}=\Xi_{2}\left(w_{i}+1\right)$.*

**Proof.**  The proof uses a similar method as in the case of iSIC in Appendix C.   □

#### 3.4.3. EC of Cell-Edge User

In this part, the EC of F is evaluated in two scenarios, NL and DL. The EC of F in case of NL can be expressed as

(43) $$\begin{array}{c} {\bar{C}}_{F}^{\mathrm{NL}}=\frac{1-\alpha }{\ln2}\int_{0}^{\Theta_{1}}\frac{1-F_{\gamma_{F}}\left(x\right)}{\left(1+x\right)}dx, \end{array}$$

where $\Theta_{1}=\min\left(\frac{1}{\beta_{1}},\frac{1}{\psi_{2}\beta_{2}}\right)$ if $\psi_{2}>0$ and $\Theta_{1}=\frac{1}{\beta_{1}}$ if $\psi_{2}=0$. Similar to the case of EC of D, the exact closed-form for EC of F in the case of NL is intricate due to the existence of the multi-summation Bessel terms and confluent hyper-geometric Kummer U function. Thus, we rely on the GCQ to solve this issue. The resulting exact closed-form approximation can be expressed in the following proposition.

**Proposition** **4.** *The closed-form analytical approximate expression for EC of F in case of NL can be attained as*

(44) C¯FNL=Ξ31−αln2∑i=1Nπ1−wi2N1+ϕ¯3iexp−Ψ¯1msnρΩsn−Ψ¯1msrΩsrρ∑ksn=0msn−11ksn!Ψ¯1msnρΩsnksn[∑ksr=0msr−1∑n=0ksrksrnmsrΩsrωρksr−nmrrΩrrmsrΓmsr+nΓmrr+nΓmsrΓmrrΨ¯1ωmsr+n−ksrUmsr+n,msr−mrr+1,mrrΨ¯1ωΩrr]∑krf=0mrf−12Ψ¯2Amsr+krf2krf!msr−1!Kmsr−krf2Ψ¯2A,

*where $\Xi_{3}=\Theta_{1}/2$, ${\bar{\phi }}_{3}^{i}=\Xi_{3}\left(w_{i}+1\right)$, ${\bar{\Psi }}_{1}=\frac{{\bar{\phi }}_{3}^{i}}{\delta_{1}-{\bar{\phi }}_{3}^{i}\left(1-\delta_{1}\right)}$, and ${\bar{\Psi }}_{2}=\frac{{\bar{\phi }}_{3}^{i}\beta_{2}}{\left(1-\psi_{2}\beta_{2}{\bar{\phi }}_{3}^{i}\right)\rho \omega \delta_{2}}$.*

From (37), the EC of F in case of DL using the SC technique can be expressed as

(45) $$\begin{array}{c} {\bar{C}}_{F}^{\mathrm{DL}}\;=\;\frac{1\;-\;\alpha }{\ln2}\left\{\begin{array}{cc} \int_{0}^{1/\beta_{1}}\frac{1-F_{\gamma_{F}}\left(x\right)}{\left(1+x\right)}dx, & \;\mathrm{for}\;\psi_{2}≅0,\\ \int_{0}^{\frac{\beta_{2}^{-1}}{\psi_{2}}}\frac{1-F_{\gamma_{F}}\left(x\right)}{\left(1+x\right)}dx\;+\;\int_{\frac{\beta_{2}^{-1}}{\psi_{2}}}^{1/\beta_{1}}\frac{1-F_{\gamma_{F}}\left(x\right)}{\left(1+x\right)}dx, & \;\mathrm{for}\;\frac{\beta_{2}^{-1}}{\psi_{2}}\;<\;\frac{1}{\beta_{1}}, \end{array}\right. \end{array}$$

where the CDF function for $\psi_{2}≅0$ is given by (31) and two CDF functions for $\frac{\beta_{2}^{-1}}{\psi_{2}}\;<\;\frac{1}{\beta_{1}}$ are given as (31) and (32), respectively.

Likewise, the CDF functions of (31) and (32) contain the multi-summation Bessel terms and confluent hyper-geometric Kummer U function. Thus, it cannot solve the exact closed-form expression for EC. To address this issue, we employ GCQ to compute a tightly bounded expression for the EC of F. The closed-form approximate solution for each case of (45) can be expressed in the following proposition.

**Proposition** **5.** *The closed-form analytical approximate expression for the EC of F in the case of DL with the first condition *(45)* can be attained as*

(46) C¯F,1DL=Ξ41−αln2∑i=1Nπ1−wi2N1+ϕ¯4iexp−Ψ¯3msnρΩsn−Ψ¯3msrΩsrρ∑ksn=0msn−11ksn!Ψ¯3msnρΩsnksn[∑ksr=0msr−1∑n=0ksrksrnmsrΩsrωρksr−nmrrΩrrmsrΓmsr+nΓmrr+nΓmsrΓmrrΨ¯3ωmsr+n−ksrUmsr+n,msr−mrr+1,mrrΨ¯3ωΩrr][∑krf=0mrf−12Ψ¯4Amsr+krf2krf!msr−1!Kmsr−krf2Ψ¯4A+exp−Ψ¯3msfρΩsf∑ksf=0msf−11ksf!Ψ¯3msfρΩsfksf1−∑krf=0mrf−12Ψ¯4Amsr+krf2krf!msr−1!Kmsr−krf2Ψ¯4A],

*where $\Xi_{4}=1/2\beta_{1}$, ${\bar{\phi }}_{4}^{i}=\Xi_{4}\left(w_{i}+1\right)$, ${\bar{\Psi }}_{3}=\frac{{\bar{\phi }}_{4}^{i}}{\delta_{1}-{\bar{\phi }}_{4}^{i}\left(1-\delta_{1}\right)}$, and ${\bar{\Psi }}_{4}=\frac{{\bar{\phi }}_{4}^{i}\beta_{2}}{\left(1-\psi_{2}\beta_{2}{\bar{\phi }}_{4}^{i}\right)\rho \omega \delta_{2}}$.*

**Proposition** **6.** *The closed-form analytical approximate expression for EC of F in case of DL with the second condition of *(45)* can be attained as*

(47) C¯F,2DL=Ξ51−αln2∑i=1Nπ1−wi2N1+ϕ¯5iexp−Ψ¯5msnρΩsn−Ψ¯5msrΩsrρ∑ksn=0msn−11ksn!Ψ¯5msnρΩsnksn[∑ksr=0msr−1∑n=0ksrksrnmsrΩsrωρksr−nmrrΩrrmsrΓmsr+nΓmrr+nΓmsrΓmrrΨ¯5ωmsr+n−ksrUmsr+n,msr−mrr+1,mrrΨ¯5ωΩrr][∑krf=0mrf−12Ψ¯6Amsr+krf2krf!msr−1!Kmsr−krf2Ψ¯6A+exp−Ψ¯5msfρΩsf∑ksf=0msf−11ksf!Ψ¯5msfρΩsfksf1−∑krf=0mrf−12Ψ¯6Amsr+krf2krf!msr−1!Kmsr−krf2Ψ¯6A]+Ξ61−αln2∑i=1Nπ1−wi2N1+ϕ¯6iexp−Ψ¯7msnρΩsn−Ψ¯7msrΩsrρ∑ksn=0msn−11ksn!Ψ¯7msnρΩsnksn[∑ksr=0msr−1∑n=0ksrksrnmsrΩsrωρksr−nmrrΩrrmsrΓmsr+nΓmrr+nΓmsrΓmrrΨ¯7ωmsr+n−ksrUmsr+n,msr−mrr+1,mrrΨ¯7ωΩrr]exp−Ψ¯7msfρΩsf∑ksf=0msf−11ksf!Ψ¯7msfρΩsfksf,

*where the parameters of the first term are $\Xi_{5}=\frac{1}{\psi_{2}\beta_{2}}$, ${\bar{\phi }}_{5}^{i}=\Xi_{5}\left(w_{i}+1\right)$, ${\bar{\Psi }}_{5}=\frac{{\bar{\phi }}_{5}^{i}}{\delta_{1}-{\bar{\phi }}_{5}^{i}\left(1-\delta_{1}\right)}$ and ${\bar{\Psi }}_{6}=\frac{{\bar{\phi }}_{5}^{i}\beta_{2}}{\left(1-\psi_{2}\beta_{2}{\bar{\phi }}_{5}^{i}\right)\rho \omega \delta_{2}}$ while the parameters of the second term are $\Xi_{6}=1/2\beta_{1}-\frac{1}{\psi_{2}\beta_{2}}$, ${\bar{\phi }}_{6}^{i}=\left(1/2\beta_{1}-\frac{1}{\psi_{2}\beta_{2}}\right)w_{i}+1/2\beta_{1}+\frac{1}{\psi_{2}\beta_{2}}$, and ${\bar{\Psi }}_{7}=\frac{{\bar{\phi }}_{5}^{i}}{\delta_{1}-{\bar{\phi }}_{6}^{i}\left(1-\delta_{1}\right)}$.*

### 3.5. Optimal Solution for the Time-Switching Factor

In TS-SWIPT NOMA-based IoT relay systems, the TS factor α has played an essential role in enhancing and improving the system performance of an adaptive system through adjustment of the EH time and information transmission processes. However, with the current forms of system throughput, it is very difficult to find the exact closed-form expression of the optimal TS value; thus, we propose a simple one-dimensional search method to solve the optimal value of α over the integral of (0,1), where the degree of accuracy of the proposed method is subject to the given step size of ϵ. In addition, the time complexity of the linear search method is also confirmed as $O\left(n\right)$ in [41], where *n* is the size of any input values. The algorithm to solve the optimal TS factor can be expressed in Algorithm 1.   
**Algorithm 1** Optimal TS factor to maximize the system throughput.  1:**function** SEARCH OPTIMAL($\alpha ,T_{\mathrm{put}}^{G}$)  2:    $T_{\mathrm{put}}^{G,\max}\leftarrow 0,i\leftarrow 1,\alpha^{\mathrm{opt}}\leftarrow 0$  3:    **while** i≤ length $\left(T_{\mathrm{put}}^{G}\right)$ **do**  4:           $T_{\mathrm{put}}^{G,\mathrm{temp}}\leftarrow T_{\mathrm{put}}\left(i\right)$  5:           **if** $T_{\mathrm{put}}^{G,\mathrm{temp}}>T_{\mathrm{put}}^{G,\max}$  6:              $T_{\mathrm{put}}^{G,\max}\leftarrow T_{\mathrm{put}}^{G,\mathrm{temp}}$  7:              $\alpha^{\mathrm{opt}}\leftarrow \alpha \left(i\right)$  8:           **end if**  9:        i←i+110:    **end while**11:**end function**

## 4. Simulation Results

In this section, we present analytical results for evaluating the performance of the proposed system, where the Monte Carlo simulation method is utilized to validate our analytical derivation in Matlab version R2020a. The key parameters used in this paper are provided in Table 1.

**Figure 3:**
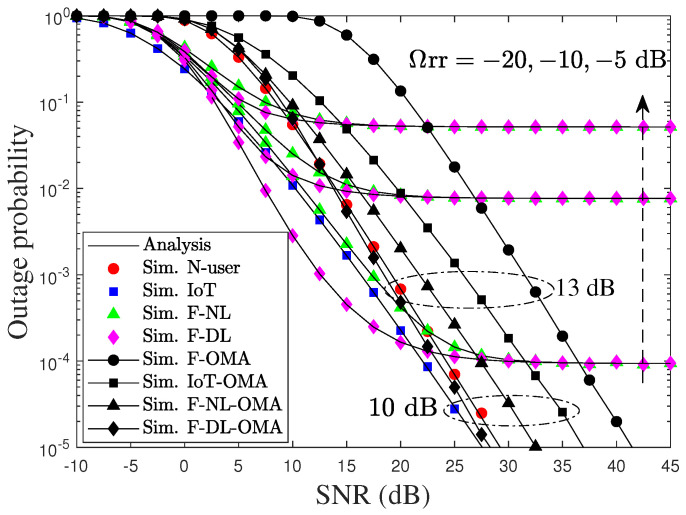
Outage performance of users versus SNR, i.e., ρ, in dB under pSIC, with $\Omega_{\mathrm{rr}}=-20,-10,-5$ dB, $m_{\mathrm{sr}}=m_{\mathrm{sn}}=m_{\mathrm{sf}}=m_{\mathrm{rd}}=m_{\mathrm{rf}}=m_{\mathrm{rr}}=2$, and a different number of truncated terms.

Figure 3 shows the OP of the user versus the average SNRs under pSIC compared to its OMA counterpart, with different values of residual SI. The N-user, IoT, and F stand for N,D, and F, respectively. First of all, it is shown that the exact analytical results perfectly match with the simulations. Moreover, the OPs of the cell-center and IoT user are better by 13 and 10 dB than those of the OMA scheme, respectively, while the performances of the faraway user with NL and DL depend on the level of $\Omega_{\mathrm{rr}}$. For example, the OPs of F are always superior to OMA at SNRs lower 22.5 dB, as the residual SI equals −20 dB. Conversely, an increase of SI decreases the OP of F as $\Omega_{\mathrm{rr}}$ increases from −20 to −10 and −5 dB. This is due to the fact that the increasing SI increases harmful noise, leading to a reduced capability decoding signal of F at R. We can also observe that the performance of F is significantly improved by adopting the SC technique for the lower SNR regime. To study the impact of the other parameter, we assumed that $\Omega_{\mathrm{rr}}$ equals −20 dB for the next investigations.

**Figure 4:**
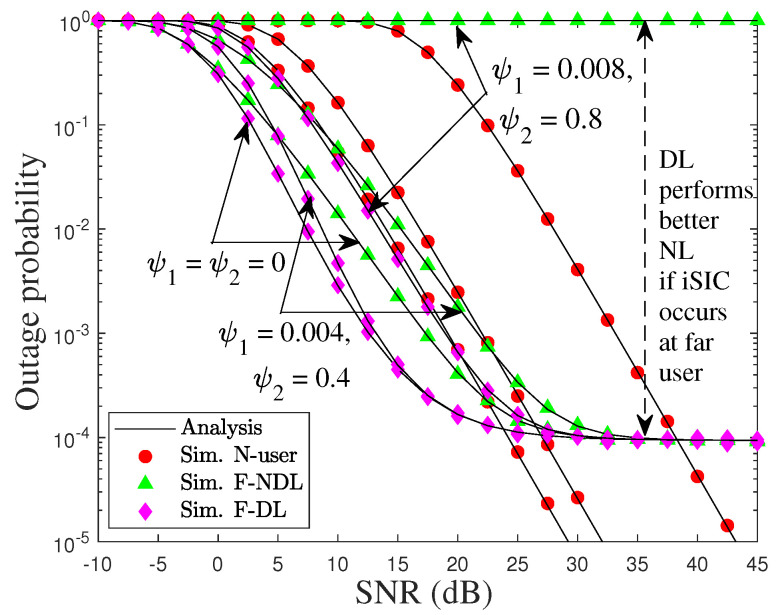
Outage performance of N and F versus SNR under iSIC, i.e., $\psi_{1}=0,0.04,0.08$ and $\psi_{2}=0,0.4,0.8$, with $\Omega_{\mathrm{rr}}=-20$ dB.

Figure 4 illustrates the impact of iSIC on the OPs of the center and edge user. It can be observed that their performances decrease as $\psi_{1}$ and $\psi_{2}$ increase. This is because both users perform SIC to decode the signals of $x_{F}$ and $x_{D}$ in the first and second NOMA communication stage, respectively. For instance, the amount of residual interference noise appears during the first SIC processes from 4% to 8%, resulting in the OP of N increasing drastically. Meanwhile, the OP of F tightly increases as iSIC increases 40%, but it significantly increased with 80%, especially where the OP of F equals 1 in the case of NL. Conversely, the OP of F maintains at a certain level by adopting the SC technique, confirming the benefits of SC for a cooperative NOMA-based IoT relay network. This is because F with DL can select the best link for the decoding signal and discard the worst link, while the F with NL is dominated by the relaying signal. From these results, we can conclude that an edge-user’s performance can further improve if the cellular network is combined with the wireless sensor network, regardless of iSIC occurrence.

**Figure 5:**
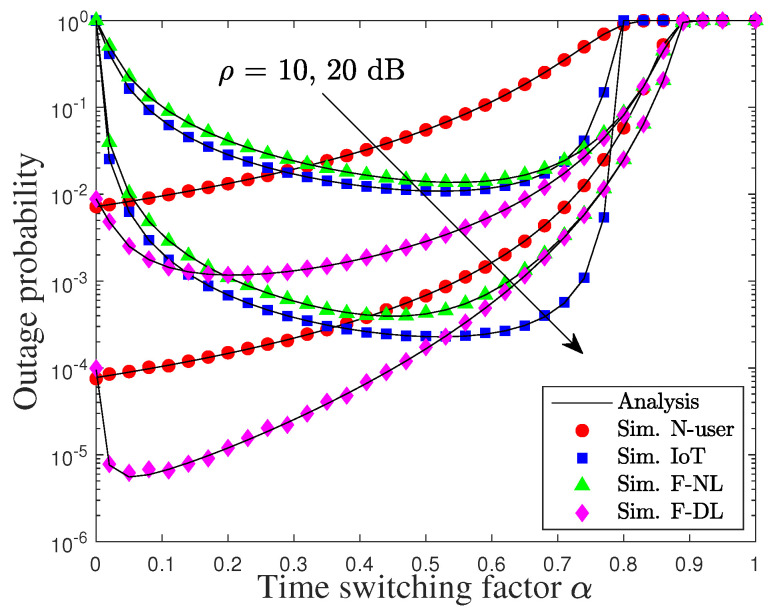
Outage performance of users versus the TS factor, i.e., ρ, under pSIC.

Figure 5 plots the OPs of users versus the TS factor α with various transmit powers. It can be seen that the OP of N is always increasing as α increases. On the other hand, the minimum OP of F and D are concave functions, and so, there exist minimal points that can achieve the minimum OP of F and D. For instance, the outage events of D and F in NL occur when α is larger than 0.8 and the outage event of F in DL occurs when α is larger than 0.9, regardless of the increase in transmit power. This is because both the source and IoT relay nodes do not have enough time for data transmission.

**Figure 6:**
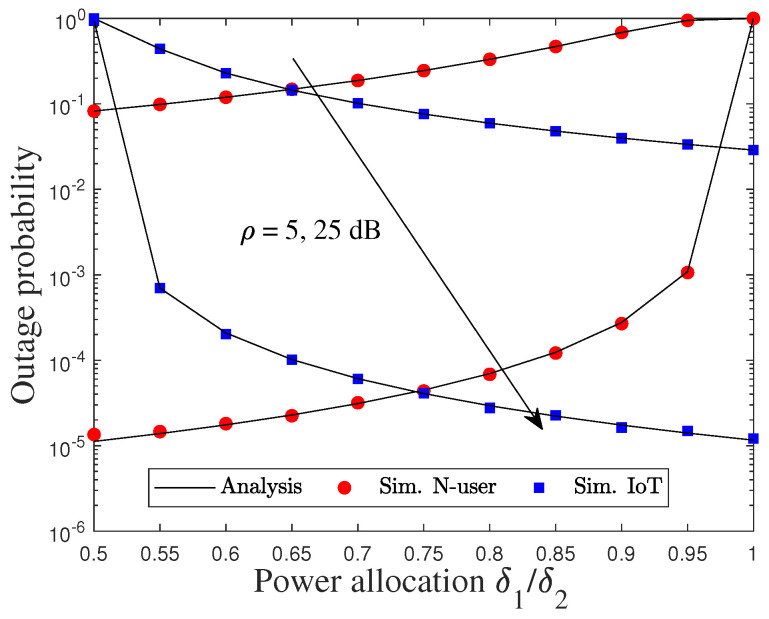
Outage performance of N and D versus the PA factor, i.e., $\delta_{1}$ and $\delta_{2}$.

**Figure 7:**
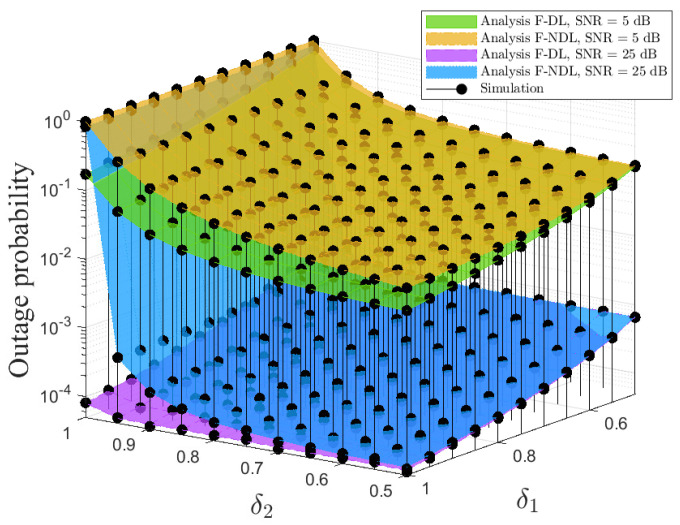
Outage performance of F versus the double PA factor, i.e., $\delta_{1}$ and $\delta_{2}$ with two dimensions.

Figure 6 and Figure 7 plot the impact of the PA factor $\delta_{1}$/$\delta_{2}$ on the user’s OPs with various average SNR levels, i.e., SNR = 5, 25 dB under pSIC. As depicted in Figure 6, the OP of N significantly increases as $\delta_{1}$ increases, and its performance cannot be improved when the average SNR increases at high $\delta_{1}$. By contrast, the OP of D dramatically decreases as $\delta_{2}$ and the average SNR increase. This is because the larger value of $\delta_{1}$ assigned to F leads to a reduced performance of N, while the larger value of $\delta_{2}$ allocated to D results in the performance degradation of F. Likewise, we can observe that the trade-off performance of F is opposite to that of N and D in Figure 7, which are almost the same via the above reasons. Interestingly, the OP of F can reduce a significant improvement at high $\delta_{1}$ and low $\delta_{2}$.

**Figure 8:**
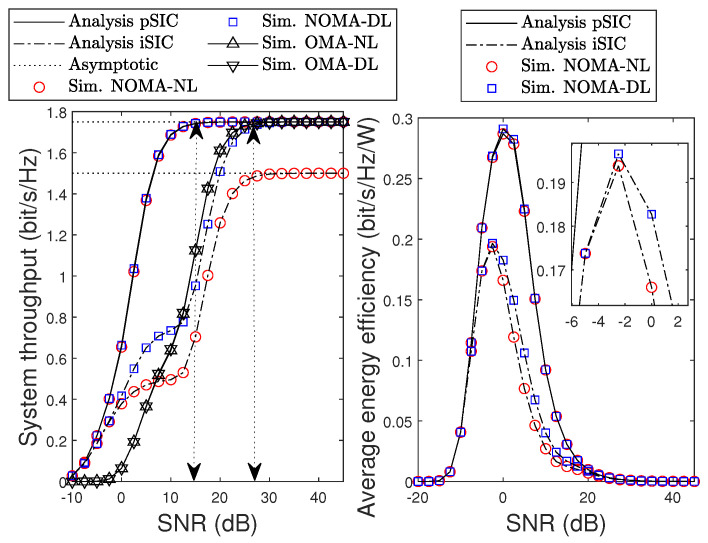
System throughput and EE versus SNR with and without iSIC, i.e., $\psi_{1}=0,0.08$, and $\psi_{2}=0,0.8$.

Figure 8 shows the system throughput and EE versus average SNR with pSIC and iSIC. As can be observed in the left-hand side of Figure 8, the considered system is outstanding compared to OMA with an SNR lower than 28 dB before approaching the convergence region under pSIC. Regarding iSIC, the results show that the system throughput performance of the NL scenario degrades significantly with $\psi_{1}=0.08$ and $\psi_{2}=0.8$, while the system throughput performance of the DL scenario is close to the bounded region with a high SNR. This is because 80% of the residual SI leads to an outage event of F during the SIC process. Consequently, the system throughput performance is also degraded. In the right-hand side of Figure 8, we show the average EE of the considered system versus SNR. It can be observed that the system EE monotonically increases as the SNR increases. However, both system EEs of pSIC and iSIC are skewed at a larger SNR, since it is now dominated by the circuit power consumption provided in (35).

**Figure 9:**
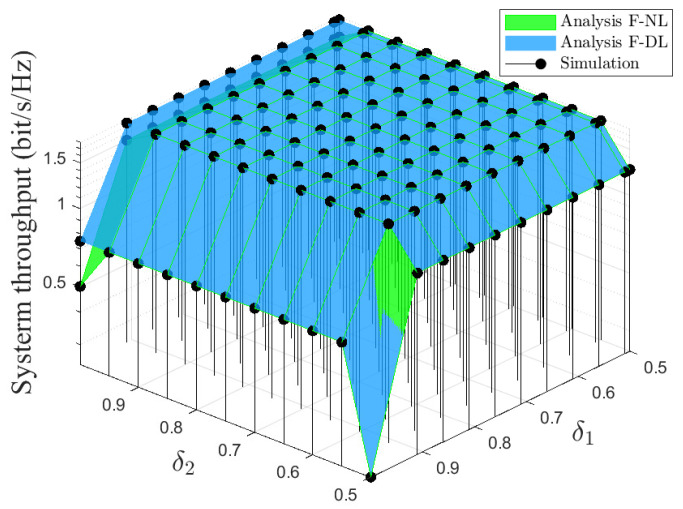
System throughput versus the double PA factor, i.e., $\delta_{1},\delta_{2}$, with SNR = 20 dB under pSIC.

Figure 9 shows the impact of the PA factors in the two communication stages on system throughput under pSIC at SNR = 20 dB. It is shown that the system throughput is balanced and reaches the bounded region with both $\delta_{1}$ and $\delta_{2}$ in the range of $\left[0,55,0,95\right]$. Hence, a pair of PA factors can be selected randomly in this range, which can always achieve maximum system throughput.

**Figure 10:**
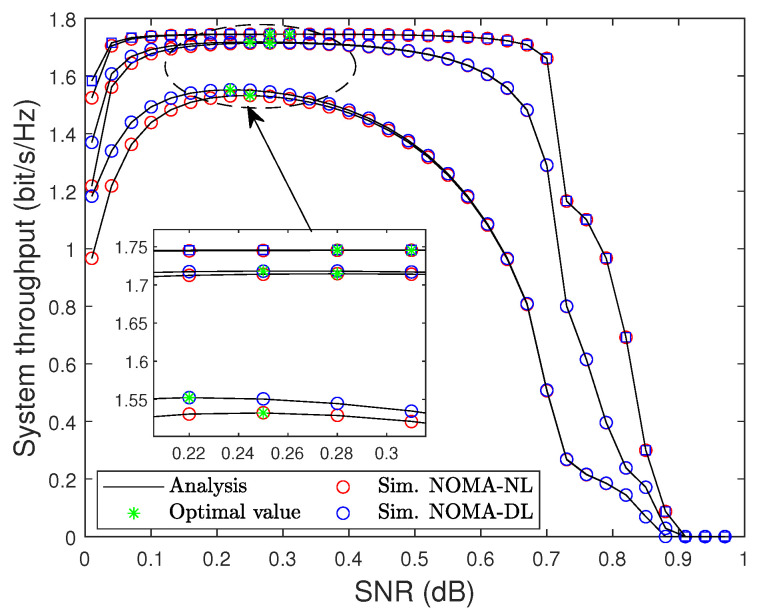
System throughput versus the TS factor α under pSIC with different SNRs, i.e., SNR = 5, 10, 15 dB.

In Figure 10, the impact of the TS factor on system throughput is investigated. The system throughput increases as α increases before hitting the maximum value; then, it degrades quickly when α is larger than 0.7. Therefore, the optimal value of α is an essential problem and should be investigated. By performing a simple one-dimensional search as presented in Algorithm 1, we can find the optimal value of α to improve the system throughput performance.

**Figure 11:**
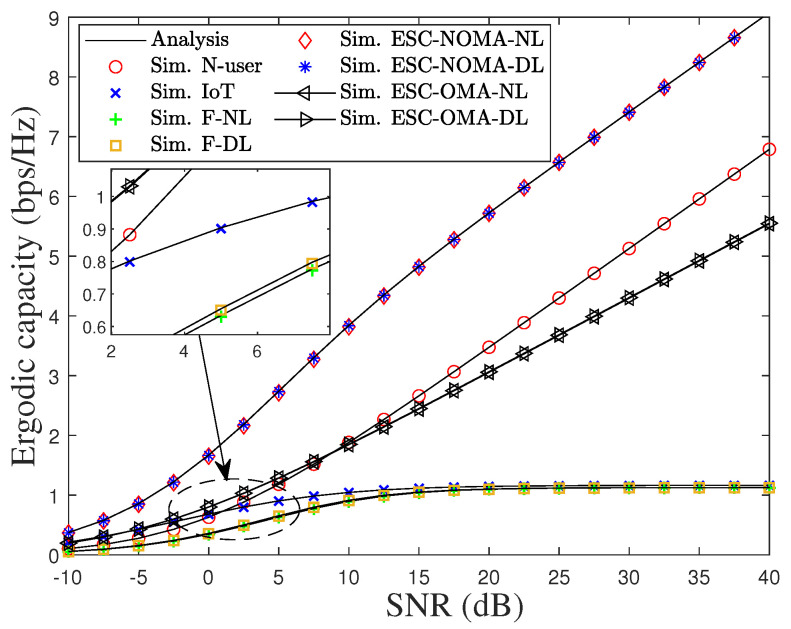
EC of the users, ESC of the considered system, and ESC of OMA scheme versus SNR under pSIC.

Figure 11 depicts the EC of the users, the ESC of the considered system, and the ESC of the OMA scheme versus SNR under pSIC. As can be observed, the EC and ESC of all schemes increase as the transmit power of the source node increases. In addition, the ESC of the proposed scheme always outperforms the OMA-based one owing to IoT communication during the cooperative transmission process. In particular, the EC of F with NL can be approximated to the EC in the case of DL, and therefore, the ESC with NL can also be approximated to the ESC with the DL scenario. This is because F in the DL scenario employs the SC technique to choose the best signal between DL and NL while F with NL is dominated by the forward signal from R.

**Figure 12:**
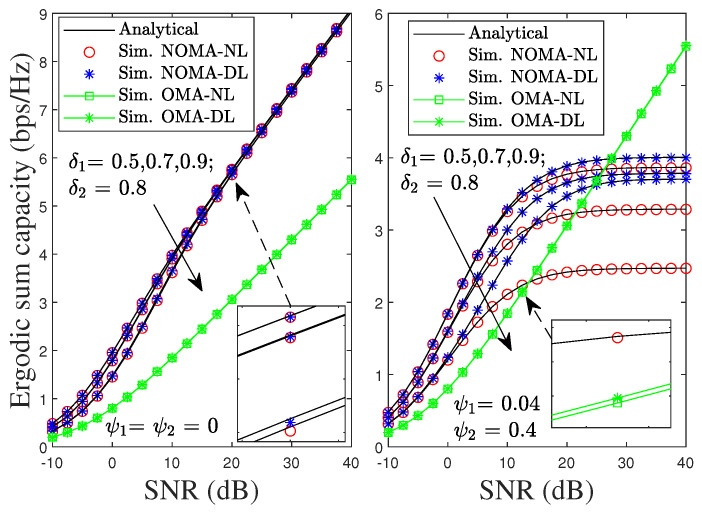
ESC of the proposed NOMA and OMA schemes versus SNR with different values of $\delta_{1}$ under pSIC and iSIC.

Figure 12 shows the impact of $\delta_{1}$ on ESC in the proposed system. The ESC of the proposed NOMA scheme in the left-hand side of Figure 12 under pSIC always outperforms the ESC of its OMA counterpart for different values of $\delta_{1}$. In addition, the ESC of the proposed NOMA scheme with the NL is approximated to DL and is not affected by the increase in $\delta_{1}$. As shown in the right-hand side of Figure 12, the proposed NOMA scheme under iSIC also achieves better ESC than its counterpart from low to medium values of SNR and worse performance at high SNR. Unlike pSIC, the ESC of the proposed NOMA scheme under iSIC is dominated by the value of $\delta_{1}$. Specifically, a higher value of $\delta_{1}$ significantly degrades the ESC of the considered NOMA-NL scenario with $\delta_{1}$ from 0.5 to 0.9, even considering the impact of the residual SI, i.e., $\psi_{1}=0.04$ and $\psi_{2}=0.4$. Fortunately, the ESC of the proposed NOMA scheme can be further improved with the DL scenario by using the SC technique.

**Figure 13:**
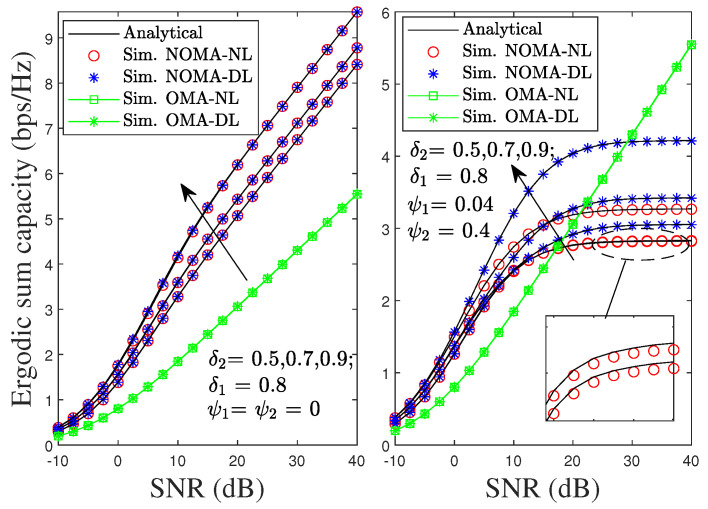
ESC of the proposed NOMA and OMA scheme versus SNR with different values of $\delta_{2}$ under pSIC and iSIC.

Figure 13 shows the impact of $\delta_{2}$ on the ESC of the proposed system. Different from $\delta_{1}$, the ESC of the proposed NOMA scheme under pSIC in the left-hand side of Figure 13 increases as $\delta_{2}$ increases. It is shown that the ESC of the proposed system is significantly better than that of the ESC of OMA and can be further enhanced at high $\delta_{2}$. A similar observation can be drawn for the ESC of the proposed NOMA scheme under iSIC from low to medium values of SNR. At high SNR, the considered scheme does not change, but there is a significant improvement with the DL scenario by adopting the SC technique.

**Figure 14:**
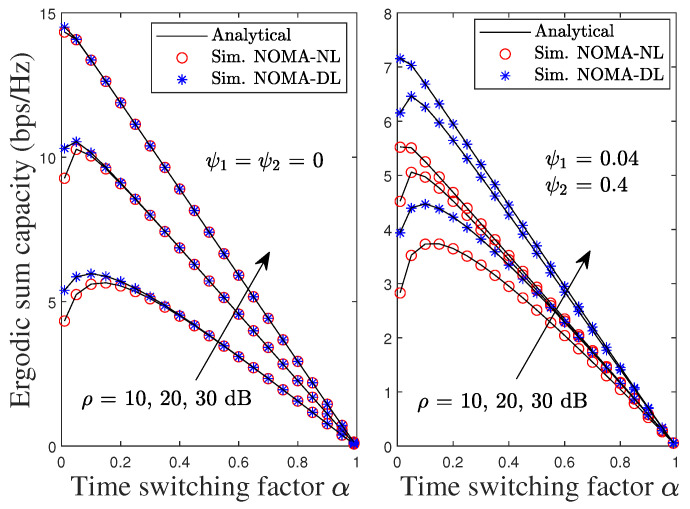
ESC of the proposed NOMA and OMA schemes versus TS factor under pSIC and iSIC.

Figure 14 illustrates the impact of α on the ESC of the proposed system. It is shown that the ESC degrades as α increases for both pSIC and iSIC. This is because a smaller α increases the information transmission, leading to the increased system capacity. It is observed that there exists a maximum point of the ESC for low and medium values of SNR, and the proposed Algorithm 1 can also provide the optimal value of α. Moreover, the results confirm that the proposed DL scenario achieves better performance than the DL scenario.

## 5. Conclusions

In this paper, we propose a novel FD SWIPT cooperative NOMA-based IoT relay network under the impact of pSIC and iSIC processes, where the SC technique is employed at a cell-edge user to improve the performance via IoT relay by adopting the NOMA protocol following two scenarios, NL and DL. Exact closed-form expressions for the OPs of users, system throughput, EE, and the approximate closed-form expressions for EC and ESC are derived and validated via the simulation method. The asymptotic expression for the system throughput is also given to provide some insights into their behaviors. In addition, to further improve the system throughput, we propose an algorithm with low complexity and high accuracy to find the optimal TS factor that guarantees maximum system throughput. Numerical results show that our proposed system is outstanding compared to its OMA counterpart in terms of the OP, system throughput, EC, and ESC under pSIC. As iSIC occurs, the performance of cell-edge users is improved significantly via the SC technique. In particular, the proposed system performance in the NL scenario degrades significantly if a high level of residual SI occurs by a cell-edge user, i.e., $\psi_{2}$ is larger than 40%. By contrast, the proposed NOMA system in the DL scenario is reduced slightly and avoids the outage event as the residual SI increases. Additionally, our proposed system shows that cellular NOMA networks can fully combine with IoT networks to solve the problems related to cell-edge users in practical development. For real applications, a system consisting of multiple users or multiple antennas will be considered in our next works. Moreover, evaluation of the privacy and security of the proposed system will be an interesting direction in the future. 

## Figures and Tables

**Table 1:** Simulation parameters.

Bandwidth	1 MHz
Antenna noise power density, $N_{0}$	−90 dBm
Fix target data rate, N	1 Bit/s/Hz
Fix target data rate of F	0.25 Bit/s/Hz
Fix target data rate of D	0.5 Bit/s/Hz
Normalized distance of S→N	0.5 unit
Normalized distance of S→F	1.4 unit
Normalized distance of S→R	0.8 unit
Normalized distance of R→F	0.6 unit
Normalized distance of R→D	0.5 unit
Path-loss exponent, θ	3
Path-loss at reference distance, L ($d_{0}$ = 1 M)	−30 dB
Fixed time switching factor, α	0.5
Fixed energy conversion efficiency, μ	0.8
Fixed total static power consumed by circuit, $P_{c}$	0.5 mW
Power allocation factors, $\delta_{1}$	0.8
Power allocation factors, $\delta_{2}$	0.8
Trial number	$10^{6}$
Fixed number of terms in the GCQ, *N*	50

## Appendix A. Proof of (27)

To derive the OP of the IoT device, invoking (4) and substituting (14) and (16) into (23), the OP of D can be expressed as

(A1) $$\begin{array}{cc} P_{\mathrm{out}}^{D}\; & =1-\mathrm{Pr}\left[\gamma_{\mathrm{rf}}^{x_{D}}\geq \gamma_{D}\right]\mathrm{Pr}\left[\gamma_{\mathrm{rd}}^{x_{D}}\geq \gamma_{D}\right]\\ \; & =1-\int_{0}^{\infty }\left[1-F_{{\left|h_{\mathrm{rf}}\right|}^{2}}\left(\frac{\gamma_{D}V_{2}}{\left(\beta_{2}-\gamma_{D}\right)x}\right)\right]\left[1-F_{{\left|h_{\mathrm{rf}}\right|}^{2}}\left(\frac{\gamma_{D}V_{2}}{\left(\beta_{2}-\gamma_{D}\right)x}\right)\right]f_{{\left|h_{\mathrm{sr}}^{\mathrm{EH}}\right|}^{2}}\left(x\right)dx, \end{array}$$

where $\beta_{2}=\frac{\delta_{2}}{1-\delta_{2}}$, $V_{2}=\frac{1}{\left(1-\delta_{2}\right)\omega \rho }$, and $\gamma_{D}<\beta_{2}$, otherwise, $P_{\mathrm{out}}^{D}=1$. By plugging $F_{{\left|h_{\mathrm{rf}}\right|}^{2}}\left(\cdot \right)$, $F_{{\left|h_{\mathrm{rd}}\right|}^{2}}\left(\cdot \right)$, and $f_{{\left|h_{\mathrm{sr}}^{\mathrm{EH}}\right|}^{2}}\left(\cdot \right)$ into (A1), the OP of D can be rewritten as

(A2) $$\begin{array}{cc} P_{\mathrm{out}}^{D}=1 & -\sum_{k_{\mathrm{rf}}=0}^{m_{\mathrm{rf}}-1}\sum_{k_{\mathrm{rd}}=0}^{m_{\mathrm{rd}}-1}\frac{A^{k_{\mathrm{rf}}}B^{k_{\mathrm{rd}}}{\left(\frac{\gamma_{D}V_{2}}{\left(\beta_{2}-\gamma_{D}\right)}\right)}^{\frac{k_{\mathrm{rf}}+k_{\mathrm{rd}}}{2}}}{k_{\mathrm{rf}}!k_{\mathrm{rd}}!\left(m_{\mathrm{sr}}-1\right)!}\\ \; & \times \int_{0}^{\infty }x^{m_{\mathrm{rf}}-k_{\mathrm{rf}}-k_{\mathrm{rd}}-1}\exp\left(-\left(\frac{m_{\mathrm{rf}}}{\Omega_{\mathrm{rf}}}+\frac{m_{\mathrm{rd}}}{\Omega_{\mathrm{rd}}}\right)\frac{\gamma_{D}V_{2}}{\left(\beta_{2}-\gamma_{D}\right)x}-\frac{m_{\mathrm{sr}}x}{\Omega_{\mathrm{sr}}}\right)dx, \end{array}$$

where $A=\frac{m_{\mathrm{rf}}m_{\mathrm{sr}}}{\Omega_{\mathrm{rf}}\Omega_{\mathrm{sr}}}$ and $B=\frac{m_{\mathrm{rd}}m_{\mathrm{sr}}}{\Omega_{\mathrm{rd}}\Omega_{\mathrm{sr}}}$. Thanks to the help of Equation (3.471.9) in [40] and after some mathematical manipulations, we can obtain the desired result as (24). The proof of Theorem 2 is complete.

## Appendix B. Proof of (29) and (31)

From (25), the OP of F in the case of NL can be rewritten as

(A3) $$\begin{array}{cc} P_{\mathrm{out}}^{F,\mathrm{NL}} & =\mathrm{Pr}\left[\min\left\{\gamma_{\mathrm{sn}}^{x_{F}},\gamma_{\mathrm{sr}}^{x_{F}},\gamma_{\mathrm{rf}}^{x_{F}}\right\}\leq \gamma_{F}\right]\\ \; & =1-\underset{I_{1}}{\underset{︸}{\mathrm{Pr}\left[\gamma_{\mathrm{sn}}^{x_{F}}\geq \gamma_{F}\right]}}\underset{I_{2}}{\underset{︸}{\mathrm{Pr}\left[\gamma_{\mathrm{sr}}^{x_{F}}\geq \gamma_{F}\right]}}\underset{I_{3}}{\underset{︸}{\mathrm{Pr}\left[\gamma_{\mathrm{rf}}^{x_{F}}\geq \gamma_{F}\right]}}. \end{array}$$

By invoking (8), we can obtain $I_{1}$ in (A3) as

(A4) $$\begin{array}{c} I_{1}=\exp\left(-\frac{\gamma_{F}{\left(1-\delta_{1}\right)}^{-1}m_{\mathrm{sn}}}{\left(1/\beta_{1}-\gamma_{F}\right)\rho \Omega_{\mathrm{sn}}}\right)\sum_{k_{\mathrm{sn}}=0}^{m_{\mathrm{sn}}-1}\frac{{\left(\frac{\gamma_{F}{\left(1-\delta_{1}\right)}^{-1}m_{\mathrm{sn}}}{\left(1/\beta_{1}-\gamma_{F}\right)\rho \Omega_{\mathrm{sn}}}\right)}^{k_{\mathrm{sn}}}}{k_{\mathrm{sn}}!}. \end{array}$$

From (4) and (10), the integral of $I_{2}$ in (A3) can be rewritten as

(A5) I2=Prhsr2>ΔωρhsrEH2hrr2+1ρ=∫x=0∞∫y=0∞1−Fhsr2Δωxy+ΔρfhsrEH2yfhrr2xdxdy=cexp−msrΔΩsrρmrrΩrrmrrmsr−1!mrr−1!∑ksr=0msr−1∑n=0ksrksrnmsrΩsrωρksr−nmsr+n−1!Δωmsr+n−ksr×∫x=0∞xmrr+n−1exp−xmrrΩrrx+1Δωmsr+ndx︸J,

where step (c) can be attained thanks to the help of Equations (1.111) and (3.351.3) in [40] and $\Delta =\frac{\gamma_{F}}{\delta_{1}-\gamma_{F}\left(1-\delta_{1}\right)}$ under the condition that $\delta_{1}-\gamma_{F}\left(1-\delta_{1}\right)>0$. With the help of Mathematica and some manipulations, the exact analytical closed-form expression for *J* in (A5) can be attained $\int_{x=0}^{\infty }\frac{x^{n}\exp\left(-\mu x\right)dx}{{\left(x+\beta \right)}^{m}}=\Gamma \left(n+1\right)\mu^{m-n-1}U\left(m,m-n,\mu \beta \right)$, where $U\left(\cdot ,\cdot ,\cdot \right)$ is the confluent hyper-geometric Kummer U function of Equation in [42] and $\Gamma \left(\cdot \right)$ presents the Gamma function of Equation (8.339) in [40]. The confluent hyper-geometric Kummer U function is also developed as a standard function in some popular mathematical software packages (i.e., Matlab, Maple, and Mathematica). By applying this formulation to (A5), we can attain the exact closed-form for $I_{2}$ into (A3) as

(A6) I2=exp−ΔmsrΩsrρ∑ksr=0msr−1∑n=0ksrksrnmsrΩsrωρksr−nmrrΩrrmsrΓmsr+nΓmrr+nΓmsrΓmrrΔωmsr+n−ksr×Umsr+n,msr−mrr+1,mrrΔωΩrr.

Next, from (4) and (15), we can attain $I_{3}$ in (A3) as

(A7) $$\begin{array}{cc} I_{3} & \;=\;\mathrm{Pr}\left({\left|h_{\mathrm{rf}}\right|}^{2}>\frac{\gamma_{F}\beta_{2}}{\left(1-\psi_{2}\beta_{2}\gamma_{F}\right)\rho \omega \delta_{2}{\left|h_{\mathrm{sr}}^{\mathrm{EH}}\right|}^{2}}\right)=\int_{x=0}^{\infty }\left[1-F_{{\left|h_{\mathrm{rf}}\right|}^{2}}\left(\frac{V_{3}}{x}\right)\right]f_{{\left|h_{\mathrm{sr}}^{\mathrm{EH}}\right|}^{2}}\left(x\right)dx\\ \; & =2\sum_{k_{\mathrm{rf}}=0}^{m_{\mathrm{rf}}-1}\frac{{\left(V_{3}A\right)}^{\frac{m_{\mathrm{sr}}+k_{\mathrm{rf}}}{2}}}{k_{\mathrm{rf}}!\left(m_{\mathrm{sr}}-1\right)!}K_{m_{\mathrm{sr}}-k_{\mathrm{rf}}}\left(2\sqrt{V_{3}A}\right), \end{array}$$

where (A7) can be obtained with the help of [40] (Equation (3.471.9)) and $V_{3}=\frac{\gamma_{F}}{\left(\delta_{2}-\psi_{2}\gamma_{F}\left(1-\delta_{2}\right)\right)\rho \omega }$ under the condition that $\delta_{2}-\psi_{2}\gamma_{F}\left(1-\delta_{2}\right)>0$. Substituting $I_{1},I_{2}$, and $I_{3}$ into (A3), we achieve the desired results as (29). The proof of Theorem 3 is complete.

From (30), the OP of F in the case of DL can be expressed as

(A8) $$\begin{array}{cc} P_{\mathrm{out}}^{F,\mathrm{DL}}=1 & -\mathrm{Pr}\left[\gamma_{\mathrm{sn}}^{x_{F}}\geq \gamma_{F}\right]\mathrm{Pr}\left[\gamma_{\mathrm{sr}}^{x_{F}}\geq \gamma_{F}\right]\left[1-\mathrm{Pr}\left[\max\left\{\gamma_{\mathrm{sf}}^{x_{F}},\gamma_{\mathrm{rf}}^{x_{F}}\right\}\leq \gamma_{F}\right]\right]\\ =1 & -I_{1}\times I_{2}\times \left[I_{3}+\underset{I_{4}}{\underset{︸}{\mathrm{Pr}\left[\gamma_{\mathrm{sf}}^{x_{F}}\geq \gamma_{F}\right]}}\left(1-I_{3}\right)\right], \end{array}$$

where $I_{4}$ in (A8) can be easily calculated as

(A9) $$\begin{array}{c} I_{4}=1-F_{{\left|h_{\mathrm{sf}}\right|}^{2}}\left[\Delta /\rho \right]=\exp\left(-\frac{\Delta m_{\mathrm{sf}}}{\rho \Omega_{\mathrm{sf}}}\right)\sum_{k_{\mathrm{sf}}=0}^{m_{\mathrm{sf}}-1}\frac{{\left(\frac{\Delta m_{\mathrm{sf}}}{\rho \Omega_{\mathrm{sf}}}\right)}^{k_{\mathrm{sf}}}}{k_{\mathrm{sf}}!}. \end{array}$$

Since $\delta_{1}-\gamma_{F}\left(1-\delta_{1}\right)>$ and $\delta_{2}-\psi_{2}\gamma_{F}\left(1-\delta_{2}\right)>0$ are the conditions that need to be satisfied according to the requirements of $I_{1},I_{2},I_{3}$, and $I_{4}$, hence, the OP of F in the case of DL can be expressed as

(A10) $$\begin{array}{c} P_{\mathrm{out}}^{F,\mathrm{DL}}=\left\{\begin{array}{cc} 1, & \mathrm{for}\;\gamma_{F}>\frac{\delta_{1}}{1-\delta_{1}},\\ 1-I_{1}I_{2}\left[I_{3}+I_{4}-I_{3}I_{4}\right], & \mathrm{for}\;C_{1},\\ 1-I_{1}\times I_{2}\times I_{4}, & \mathrm{for}\;C_{2}. \end{array}\right. \end{array}$$

where $C_{1}$ is the condition $\gamma_{F}<\min\left(\frac{\delta_{1}}{1-\delta_{1}},\frac{1}{\psi_{2}\beta_{2}}\right)$ with $\psi_{2}\in \left[0,1\right)$ and $C_{2}$ denotes the condition $\frac{1}{\psi_{2}\beta_{2}}<\gamma_{F}<\frac{\delta_{1}}{1-\delta_{1}}$ with $\psi_{2}\in \left(0,1\right)$. From (A10), we can obtain the desired results as (31) and (32) corresponding to C1 and C2, respectively. The proof of Theorem 4 is complete.

## Appendix C. Proof of (39) and (40)

From (25) and (37), we can express the EC of N in the case of pSIC as

(A11) $$\begin{array}{c} {\bar{C}}_{N}^{\mathrm{pSIC}}=\frac{1-\alpha }{\ln2}\sum_{k_{\mathrm{sn}}=0}^{m_{\mathrm{sn}}-1}\frac{{\left(V_{1}/\beta_{1}\right)}^{k_{\mathrm{sn}}}}{k_{\mathrm{sn}}!}\int_{0}^{\infty }\frac{x^{k_{\mathrm{sn}}}\exp\left(-\frac{xV_{1}}{\beta_{1}}\right)}{\left(1+x\right)}dx. \end{array}$$

Thanks to the help of Equation (3.353.5) in [40], we can obtain the desired results in (39). The proof of Proposition 1 is complete. The EC of N in the case of iSIC is formulated as

(A12) $$\begin{array}{c} {\bar{C}}_{N}^{\mathrm{iSIC}}\;=\;\frac{1-\alpha }{\ln2}\sum_{k_{\mathrm{sn}}=0}^{m_{\mathrm{sn}}-1}\frac{1}{k_{\mathrm{sn}}!}\int_{0}^{\frac{1-\delta_{1}}{\psi_{1}\delta_{1}}}\frac{\exp\left(-\frac{xV_{1}}{\beta_{1}-x\psi_{1}}\right){\left(\frac{xV_{1}}{\beta_{1}-x\psi_{1}}\right)}^{k_{\mathrm{sn}}}}{\left(1+x\right)}dx. \end{array}$$

Since the EC of N in the case of iSIC in its current form is rather intricate, we thus rely on the GCQ approximation in Equation (25.4.30) of [42], which yields

(A13) $$\begin{array}{c} \int_{a}^{b}f\left(x\right)=\frac{b-a}{2}\sum_{i=1}^{N}\frac{\pi \sqrt{1-w_{i}^{2}}}{N}f\left(\phi_{i}\right), \end{array}$$

where $w_{i}=\cos\left(\frac{\pi (2i-1)}{2N}\right)$, $\phi_{i}=\frac{b-a}{2}w+\frac{b+a}{2}$, and *N* represents the number of terms in the GCQ. By performing the GCQ for (A12) and after some derivation steps, we attain the desired results in (40). The proof of Proposition 2 is complete.
